# Supplementary material for: Systematic investigation of chemo-immunotherapy synergism to shift anti-PD-1 resistance in cancer
Source: Nat Commun. 2024 Apr 12;15:3178. doi: 10.1038/s41467-024-47433-y (PMC11015024; doi:10.1038/s41467-024-47433-y)
Supplement: Supplementary file 1 — Supplementary Information [file 41467_2024_47433_MOESM1_ESM.pdf]

Supplementary information

for

Systematic investigation of chemo-immunotherapy synergism to shift anti-PD-1 resistance in cancer

Authors: Yue Wang[1]†, Dhamotharan Pattarayan[1]†, Haozhe Huang[1]†, Yueshan Zhao[1], Sihan Li[1], Yifei Wang[1], Min Zhang[1], Song Li[1], Da Yang[1,2,3,*]

Affiliations:

[1] Center for Pharmacogenetics, Department of Pharmaceutical Sciences, University of Pittsburgh, PA 15261, USA

[2] UPMC Hillman Cancer Institute, University of Pittsburgh, Pittsburgh, PA 15261, USA

[3] Department of Computational and Systems Biology, University of Pittsburgh, Pittsburgh, PA, 15261, USA

*Corresponding author. Email: dyang@pitt.edu

† These authors contributed equally to this work.

Supplementary Figure 1

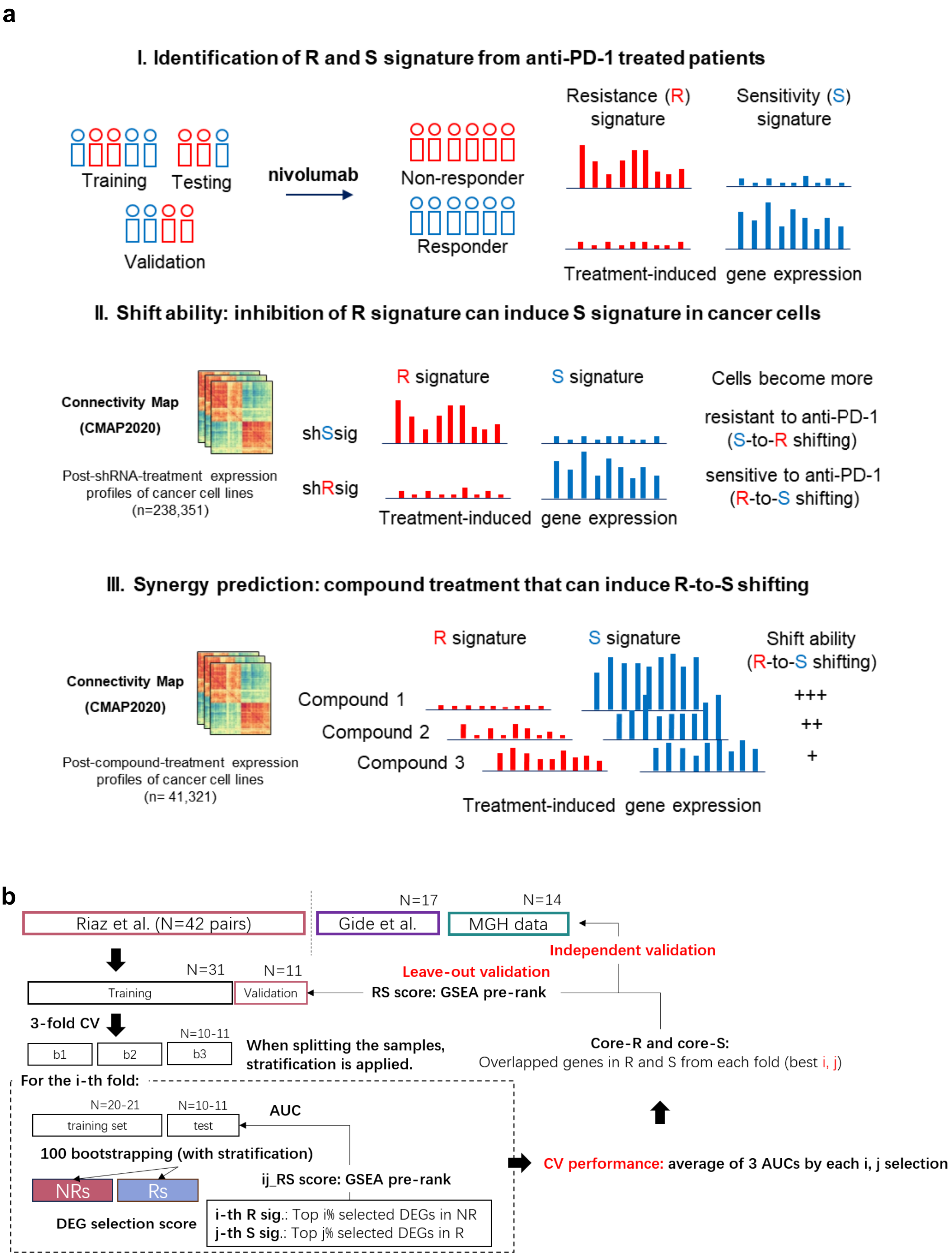

Supplementary Figure 1 (Cont'd)

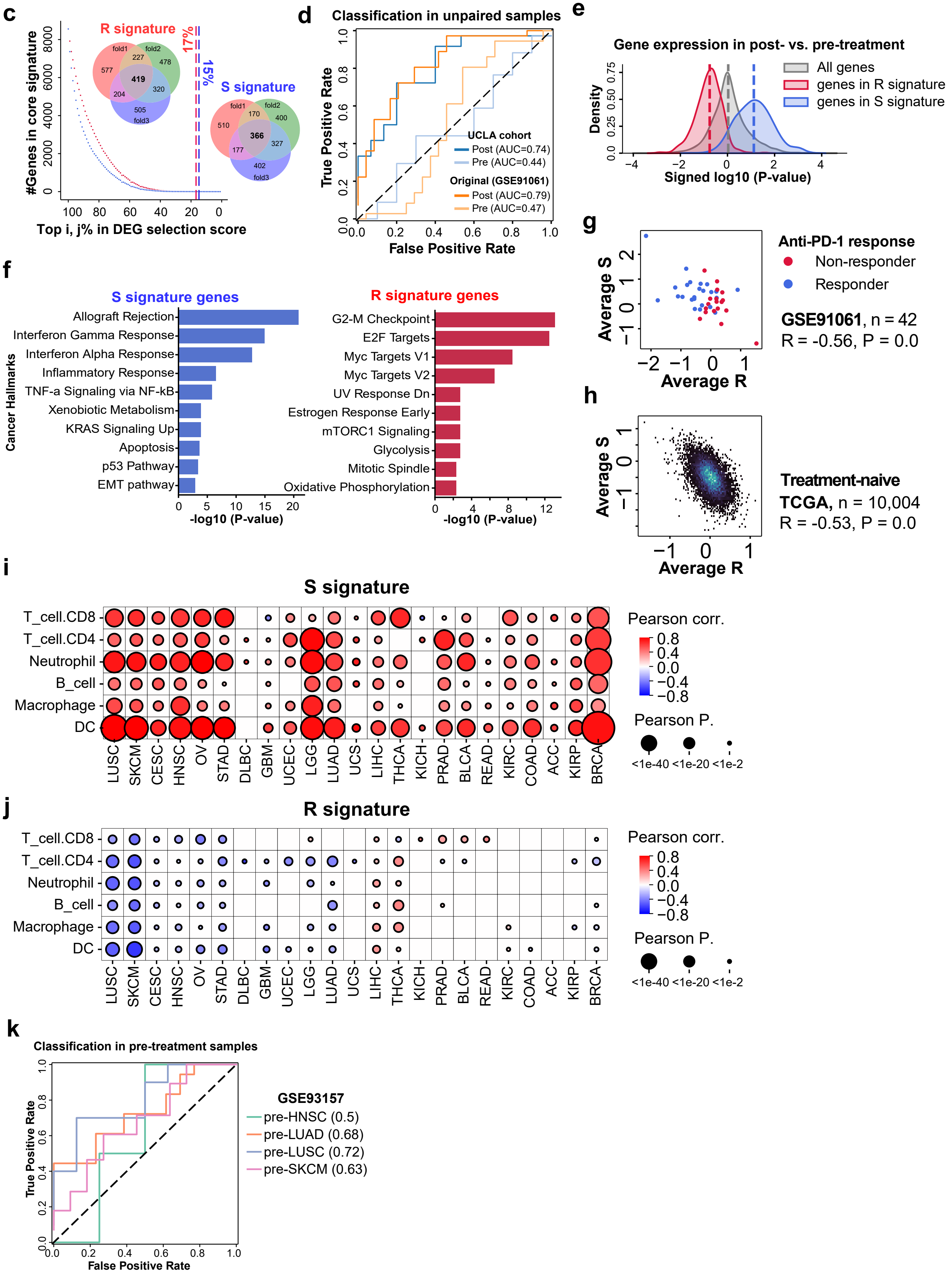

Supplementary Fig. 1. R signature and S signature genes are associated with anti-PD-1 and immune response in patients (Corresponding to Fig. 1).

a, Schematic of data analysis framework in this study. **b**, Flowchart of signature training procedure implemented in this study. **c**, Number of genes in the final Resistance (R) signature and Sensitivity (S) signature. **d**, Receiver operating characteristic (ROC) curve summarizing the performance of using R and S signatures to classify anti-PD-1 responders and non-responders in patient cohorts with unpaired samples. UCLA pre-SKCM, n = 27; UCLA post-SKCM, n = 33. **e**, Differential expression of R (red) or S (blue) genes in post-treatment samples compared to pre-treatment baselines. **f**, Cancer Hallmarks: Pathway enrichment of genes involved in S (left) or R (right) signature. X-axis represents adjusted P-value derived from gene set enrichment analysis. **g**, Association between R signature expression and S signature expression in anti-PD-1 treated patients (n = 42). **h**, Association between R signature expression and S signature expression in TCGA treatment-naïve patients (n = 10,004). **i**, Association between S signature expression and immune infiltration in TCGA cohorts (ACC, n = 79; BLCA, n = 411; BRCA, n = 1,097; CESO, n = 304; COAD, n = 467; DLBC, n = 48; GBM, n = 154; HNSC, n = 500; KICH, n = 65; KIRP, n = 288; LGG, n = 510; LIHC, n = 371; LUAD, n = 524; LUSC, n = 501; OV, n = 374; PRAD, n = 498; READ, n = 166; SKCM, n = 367; STAD, n = 375; THCA, n = 502; UCEC, n = 547; UCS, n = 56). Colormap represents correlation coefficient given by Pearson's correlation. The size of the dot represents the corresponding negative log10-transformed p-value. **j**, Association between R signature expression and immune infiltration in TCGA cohorts (ACC, n = 79; BLCA, n = 411; BRCA, n = 1,097; CESO, n = 304; COAD, n = 467; DLBC, n = 48; GBM, n = 154; HNSC, n = 500; KICH, n = 65; KIRP, n = 288; LGG, n = 510; LIHC, n = 371; LUAD, n = 524; LUSC, n = 501; OV, n = 374; PRAD, n = 498; READ, n = 166; SKCM, n = 367; STAD, n = 375; THCA, n = 502; UCEC, n = 547; UCS, n = 56). Colormap represents correlation coefficient given by Pearson's correlation. The size of the dot represents the corresponding negative log10-transformed p-value. **k**, Receiver operating characteristic (ROC) curve summarizing the performance of using R and S signatures to classify anti-PD-1 responders and non-responders in patient cohorts with unpaired samples. In GSE93157, all the patients are before treatment (i.e., treatment-naïve). HNSC: head and neck carcinoma, n = 5. LUAD: lung adenocarcinoma, n = 22. LUSC: lung squamous cell carcinoma, n = 13. SKCM: melanoma, n = 25. All the patients are treated with immune checkpoint blockades, including stand-alone anti-PD-1, or combined with anti-CTLA4.

Supplementary Figure 2:

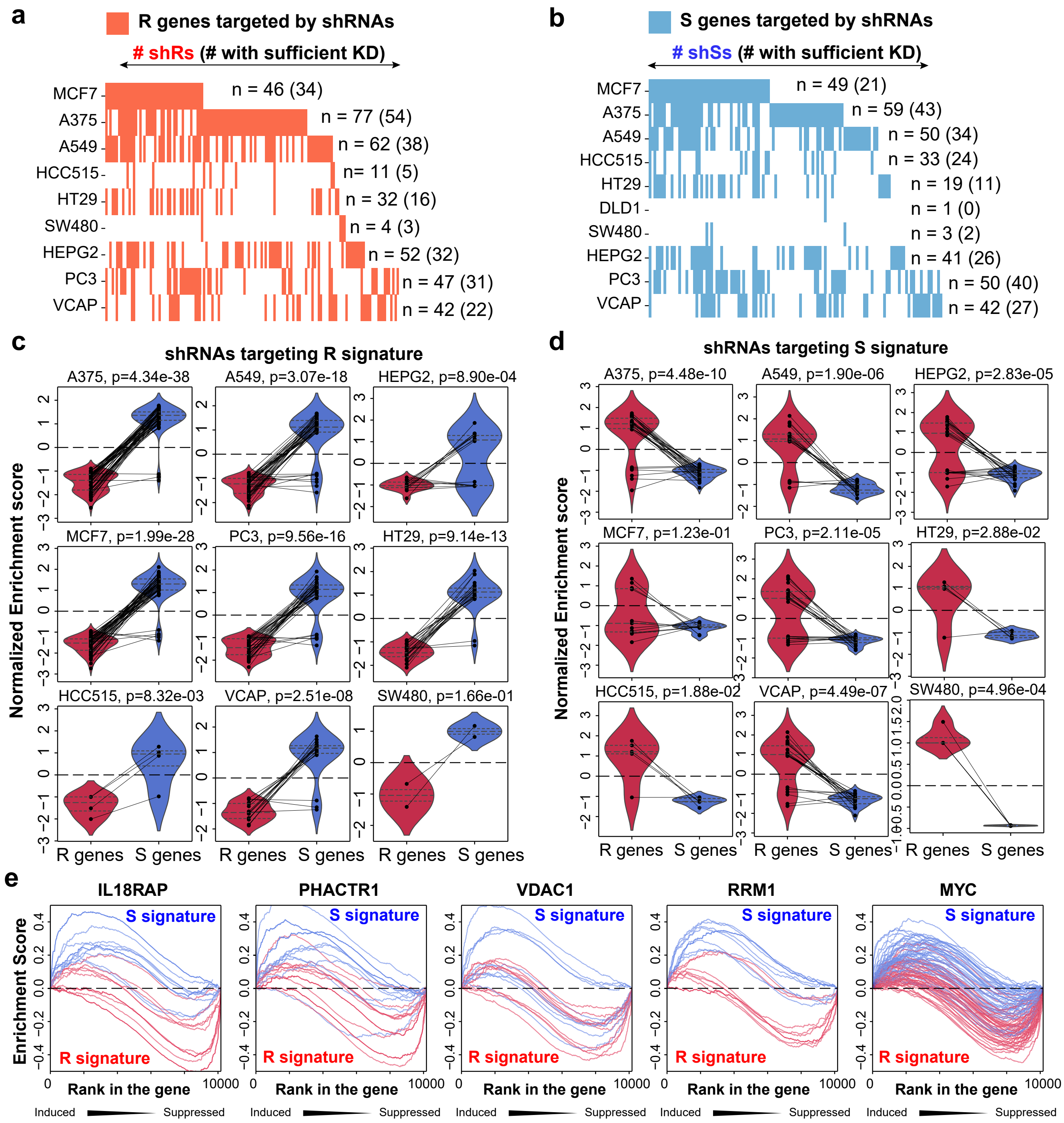

Supplementary Fig. 2. shRNAs targeting Resistance signature can induce Sensitivity signature gene expression in cancer cells (Corresponding to Fig. 2).

a, Number of R-signatures targeting shRNAs with sufficient knockdown efficiency in different cell lines. **b**, Number of S-signatures targeting shRNAs with sufficient knockdown efficiency in different cell lines. **c**, Normalized enrichment score of R signature and S signature in the cell lines after R signature genes are being knocked down. Enrichment scores coming from the same experiment are connected by the lines. *P* values are given by paired t test. **d**, Normalized enrichment score of R signature and S signature in the cell lines after S signature genes are being knocked down. Enrichment scores coming from the same experiment are connected by the lines. *P* values are given by paired t test. **e**, Enrichment curves of R signature and S signature in IL18RAP, PHACTR1, VDAC1, RRM1, and MYC knockdown cell lines.

Supplementary Figure 3:

a

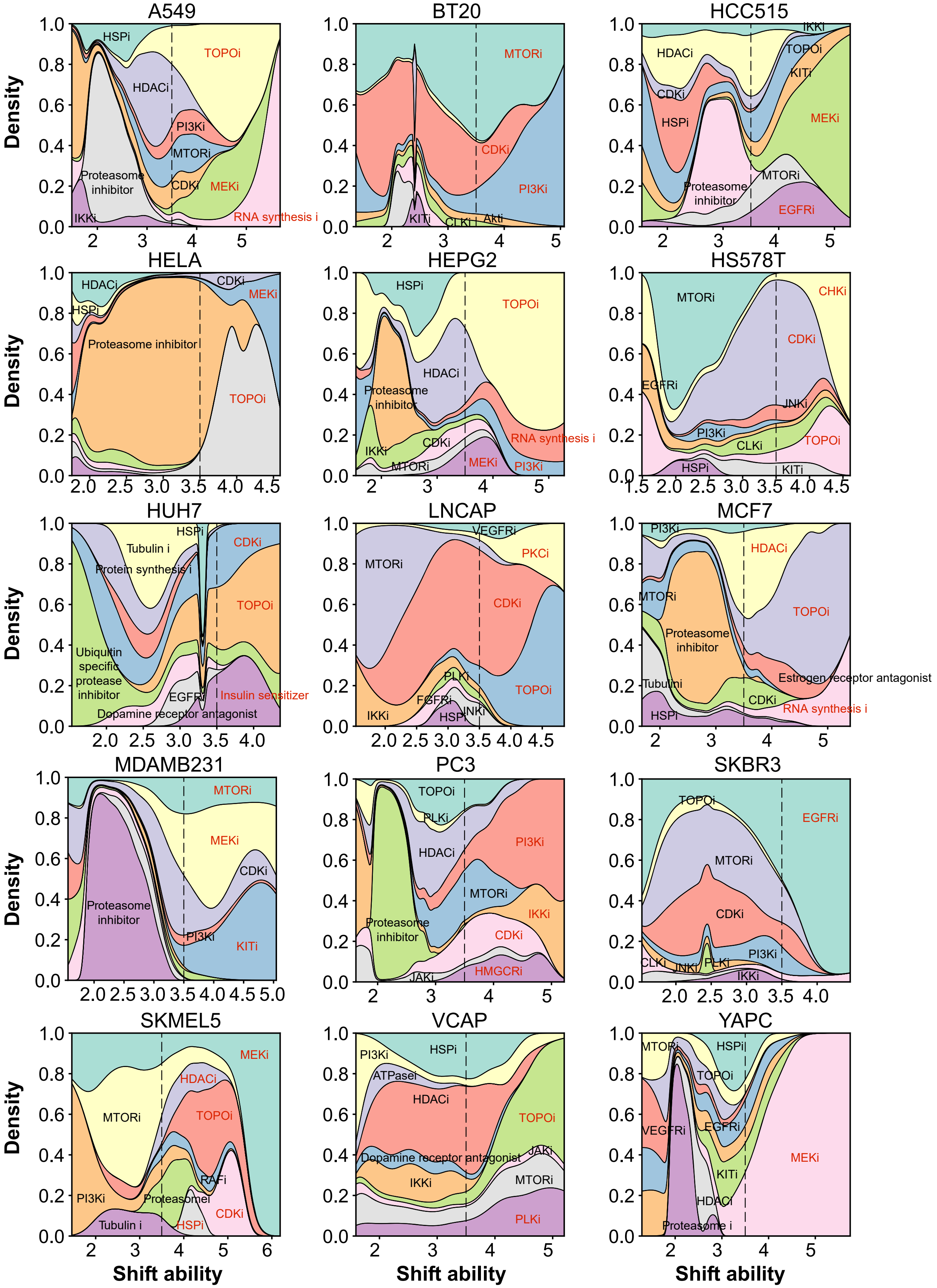

Supplementary Figure 3: (Cont'd)

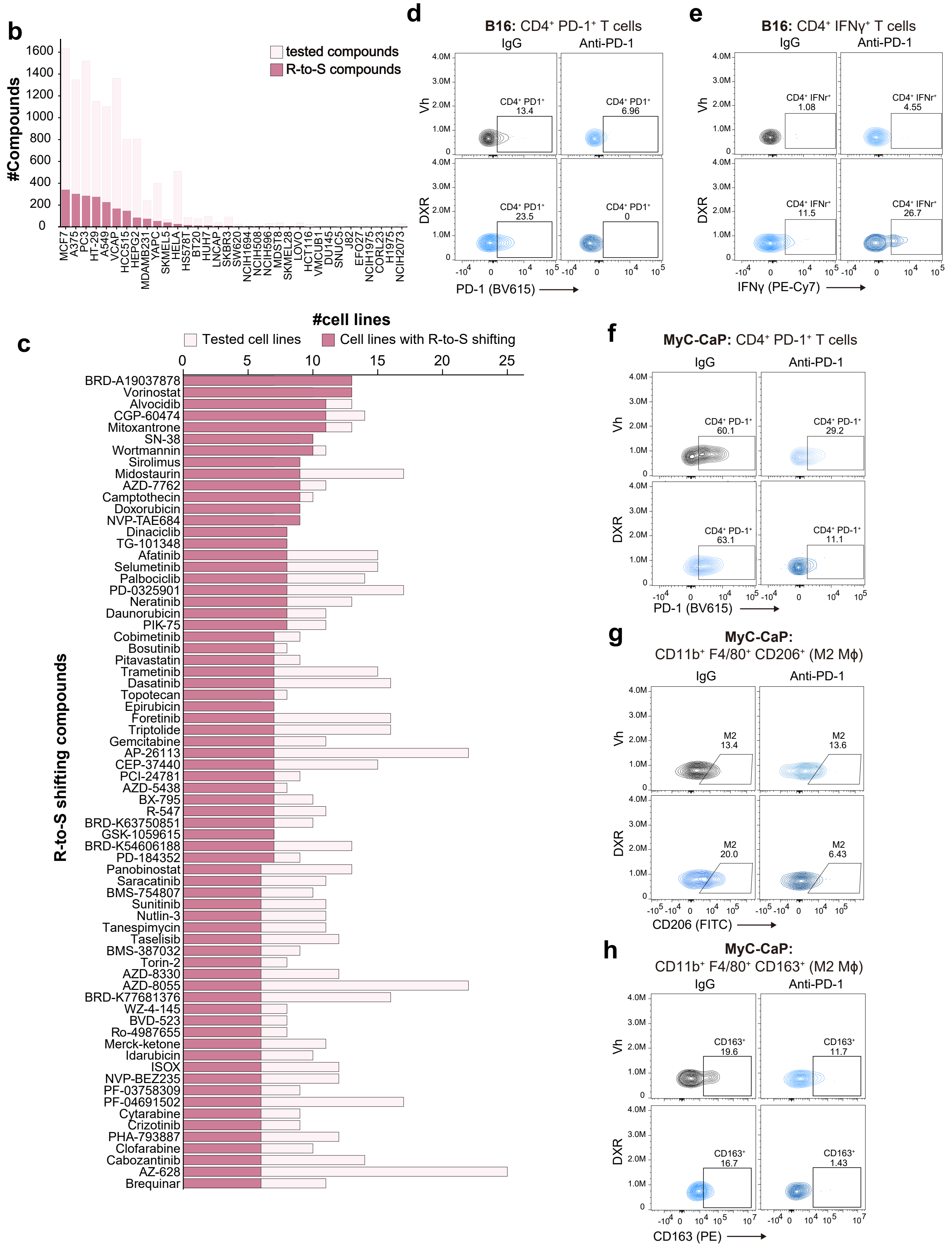

Supplementary Fig. 3. The landscape of chemo-immunotherapy synergism (Corresponding to Fig. 3).
a, Stacked density plot of top R-to-S shifting drug targets in selected cell line. X-axis indicates shift ability. The Y-axis indicates density. Red-highlighted text indicates the major drug targets in significant R-to-S shifting range (shift ability >= 3.5). **b,** Number of R-to-S shifting compounds across different cell lines. Dark pink colored bars indicate the number of R-to-S shifting compounds. Light pink bars indicate the total number of tested compounds. **c**, Number of cell lines across different compounds. Dark pink colored bars indicate the number of cell lines showing R-to-S shifting. Light pink bars indicate the total number of tested cell lines. **d**, **e**, Representative images of infiltrated $CD4^+$ $PD\text{-}1^+$ T cells (**d**) and $CD4^+$ $IFN\gamma^+$ T cells (**e**) in B16 melanoma (*n* = 5 mice). **f-h**, Representative images of infiltrated $CD4^+$ $PD\text{-}1^+$ T cells (**f**), $CD206^+$ macrophages (**g**) and $CD163^+$ macrophages (**h**) in MyC-CaP prostate cancer (*n* = 6 mice).

Supplementary Figure 4:

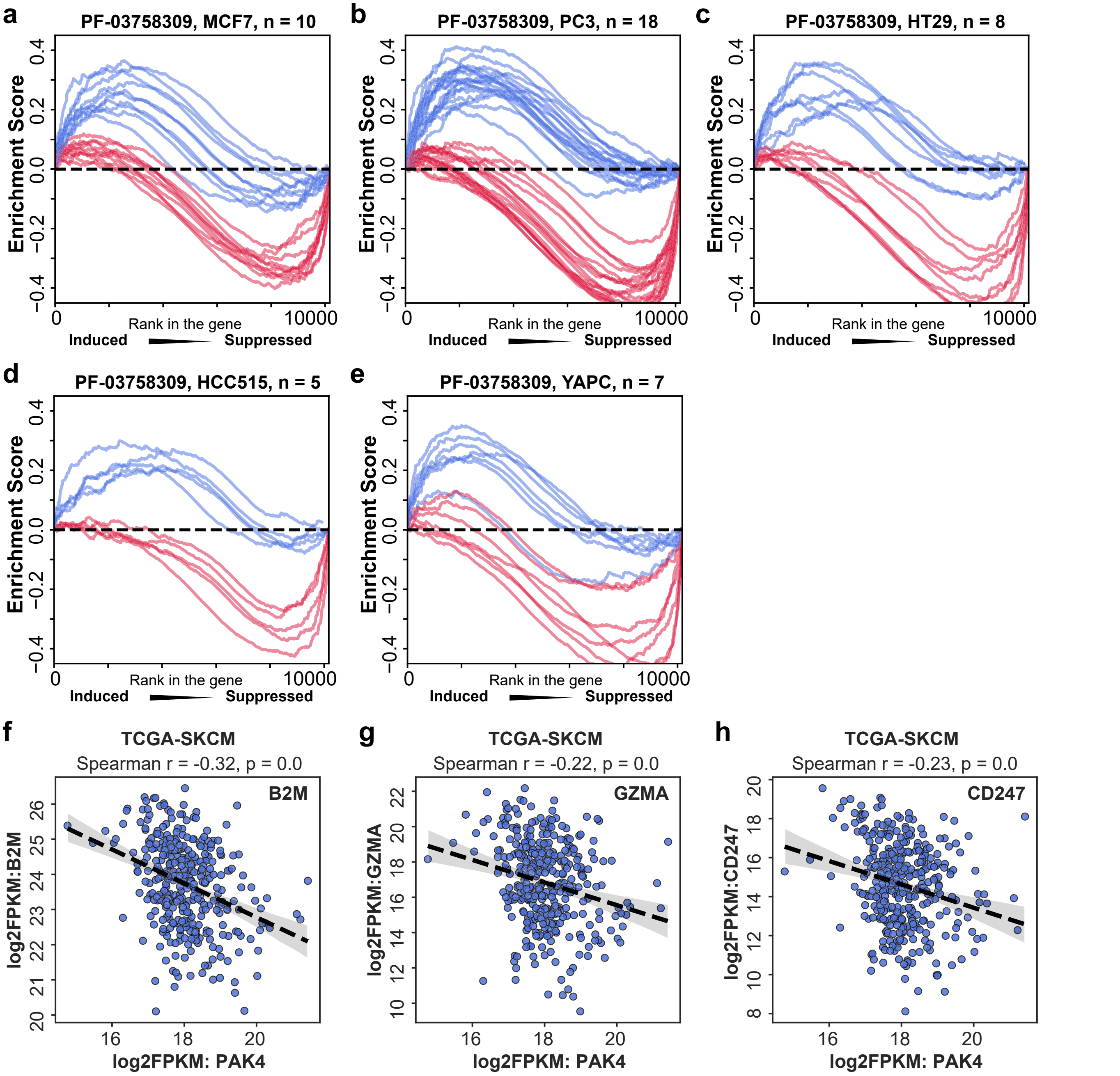

Supplementary Fig. 4. PAK4 as a potent target for chemo-immunotherapy synergism (Corresponding to Fig. 4).

a-e, Enrichment curves of R signature and S signature in PAK4 inhibitor treated cell lines (MCF7, PC3, HT29, HCC515 and YAPC). **f-h,** Association between PAK4 gene expression and B2M, GZMA and CD247 in TCGA melanoma cohorts (*n* = 367).

Supplementary Figure 5:

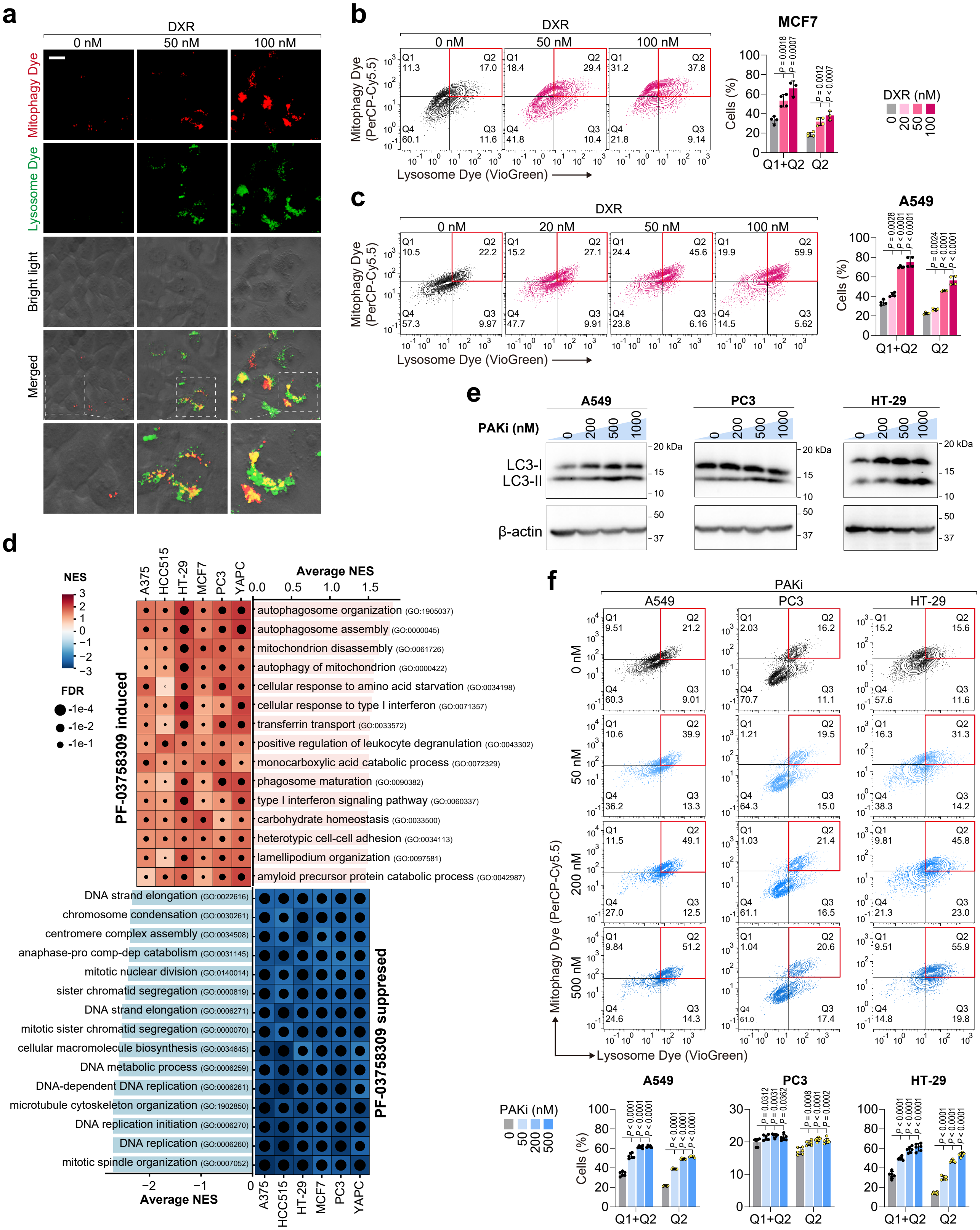

Supplementary Figure 5: (Cont'd)

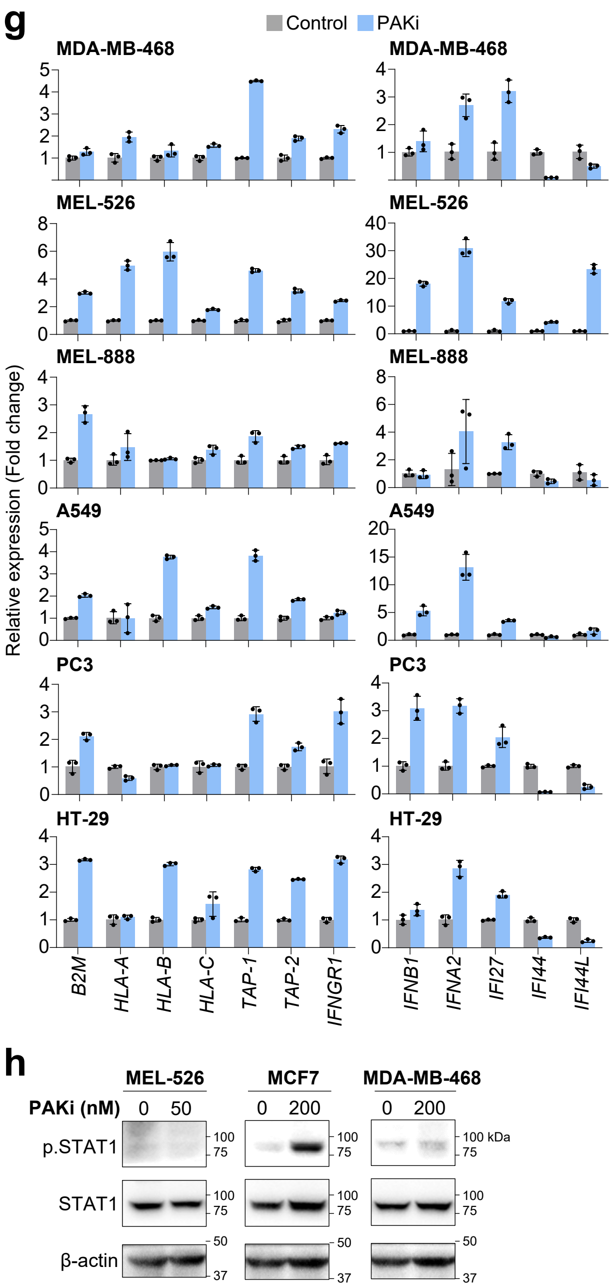

Supplementary Fig. 5. PAK inhibitor can induce mitophagy and immune response in cancer cells. **a-c**, DXR treatment induces mitophagy in MCF7 and A549 cells. **a**, Representative florescence microscopic images of MCF7 (24 h) cells labeled with mitophagy and lysosome dye, scale bar: 20 μm. **b**, **c**, Flow cytometry detection of mitophagy in MCF7 (**b**) and A549 (**c**) cells (24 h), *n* = 3-4 biologically independent samples. **d**, Top 15 pathways that are induced (red) and suppressed (blue) by PF-03758309 treatment in multiple cancer cell lines. Heatmap showed the normalized enrichment score of gene ontology pathways in corresponding cancer cell lines. The bar plot on the side of the heatmap indicates the average normalized enrichment score across different cell lines. **e**, Immunoblotting analysis of LC3 protein in cancer cells after 48 h of PAKi treatment. Experiments were repeated twice and obtained similar results. **f**, Flow cytometry detection of mitophagy in A549, PC3 and HT-29 cells (24 h), *n* = 6 biologically independent samples. **g**, qPCR analysis of antigen presenting, processing genes (left panel) and interferon stimulated genes (right panel) in cancer cells after 48 h of PAKi treatment. Concentration of PAKi used: 200 nM for MDA-MB-468 and MEL-888; 50 nM for MEL-526; 500 nM for A549, PC3 and HT-29 cells. DMSO served as control. *n* = 3 technical replicates. **h**, Immunoblotting analysis after 48 h of PAKi treatment. Experiments were repeated twice and obtained similar results. Data in **b**, **c**, **f**, and **g** are presented as mean ± SD, *P* values in **b**, **c**, and **f** were generated using a two-tailed Student's t-test. Source data are provided as a Source Data file.

Supplementary Figure 6:

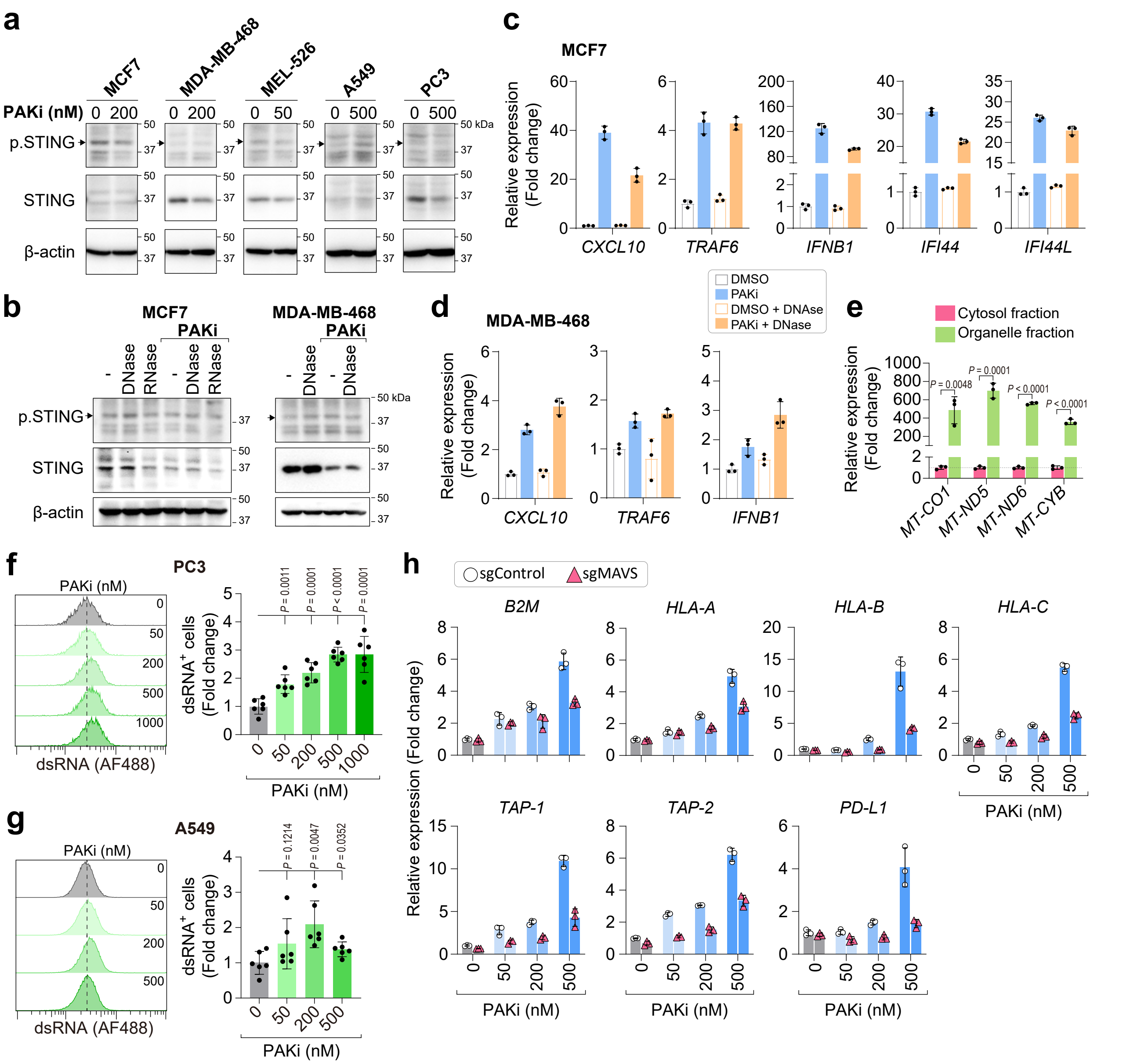

Supplementary Fig. 6. PAK4 inhibitor-induced immune responses are mediated by mtRNA-dsRNA-MAVS.

a, Immunoblotting analysis of STING in cancer cells were treated with PAKi for 48 h. Arrowheads indicate the appropriate location of p.STING. Experiments were repeated twice and obtained similar results. **b**, Immunoblotting analysis of STING in MCF7 and MDA-MB-468 cells were treated with PAKi (300 or 200 nM) for 48 h in presence of DNase or RNase. Experiments were repeated once and obtained similar results. **c**, **d**, qPCR analysis in MCF7 (**c**) and MDA-MB-468 (**d**) cells after 48 h of PAKi (300 or 200 nM) treatment in presence of DNase, $n = 3$ technical replicates. **e**, qPCR analysis indicates the expression of mitochondrial genes in fractions from MCF7 cells, ensuring purity of fractions, $n = 3$ biologically independent samples, data normalized to *GAPDH*. **f**, **g**, Flow cytometry analysis of dsRNA in PC3 (**f**) and A549 (**g**) cells after 24 h treatment, $n = 6$ biologically independent samples. **h**, qPCR analysis of antigen presenting, processing genes and *PD-L1* in MCF7 sgControl and sgMAVS cells after 48 h of PAKi treatment, $n = 3$ technical replicates. Data in **c-h** are presented as mean ± SD, P values in **e-g** were generated using a two-tailed Student's t-test. Source data are provided as a Source Data file.

Supplementary Table 1. qRT-PCR of antigen presentation genes and *PD-L1* after 48 h of treatment. n = 3 technical replicates.

PAKi	0 nM		2 or 50 nM		50 or 200 nM		500 nM	
**Gene**	**FC**	**±SD**	**FC**	**±SD**	**FC**	**±SD**	**FC**	**±SD**
**MCF7 (PAKi: 0, 50, 200, 500 nM)**								
B2M	**1.02**	0.33	**2.13**	0.19	**3.45**	0.11	**5.78**	0.20
HLA-A	**1.02**	0.36	**1.61**	0.15	**2.58**	0.10	**5.68**	0.29
HLA-B	**1.01**	0.22	**0.98**	0.08	**2.83**	0.19	**17.65**	0.26
HLA-C	**1.02**	0.34	**1.39**	0.19	**2.41**	0.02	**6.27**	0.40
TAP-1	**1.01**	0.30	**2.79**	0.34	**4.47**	0.17	**11.54**	0.57
TAP-2	**1.01**	0.26	**2.35**	0.05	**3.50**	0.23	**6.39**	0.26
IFNGR1	**1.02**	0.39	**2.07**	0.26	**2.65**	0.14	**3.27**	0.18
PD-L1	**1.01**	0.25	**0.99**	0.14	**1.38**	0.05	**3.62**	0.25
**MDA-MB-468 (PAKi: 0, 50, 200, 500 nM)**								
B2M	**1.00**	0.14	**1.17**	0.06	**1.28**	0.16	**1.06**	0.16
HLA-A	**1.01**	0.30	**1.55**	0.18	**1.96**	0.22	**0.84**	0.18
HLA-B	**1.00**	0.17	**1.37**	0.00	**1.32**	0.27	**1.08**	0.12
HLA-C	**1.00**	0.17	**1.26**	0.14	**1.57**	0.08	**1.37**	0.10
TAP-1	**1.00**	0.03	**4.37**	0.32	**4.50**	0.03	**3.37**	1.27
TAP-2	**1.01**	0.19	**1.76**	0.01	**1.90**	0.11	**1.62**	0.10
IFNGR1	**1.00**	0.05	**1.89**	0.20	**2.31**	0.17	**1.61**	0.22
PD-L1	**1.03**	0.41	**3.02**	0.37	**6.70**	0.38	**5.31**	0.63
**MEL-526 (PAKi: 0, 2, 50, 500 nM)**								
B2M	**1.00**	0.08	**1.42**	0.02	**2.99**	0.08	**3.27**	0.22
HLA-A	**1.00**	0.07	**1.46**	0.03	**4.98**	0.33	**5.16**	0.22
HLA-B	**1.00**	0.06	**1.69**	0.20	**5.97**	0.66	**2.21**	0.29
HLA-C	**1.00**	0.06	**1.13**	0.02	**1.79**	0.06	**1.47**	0.06
TAP-1	**1.00**	0.12	**1.37**	0.03	**4.62**	0.13	**6.49**	0.36
TAP-2	**1.00**	0.15	**1.52**	0.04	**3.15**	0.14	**2.51**	0.15
IFNGR1	**1.00**	0.07	**1.17**	0.08	**2.43**	0.07	**3.74**	0.08
PD-L1	**1.00**	0.16	**1.04**	0.10	**2.32**	0.44	**2.80**	0.29
**MEL-888 (PAKi: 0, 50, 200, 500 nM)**								
B2M	**1.00**	0.11	**2.54**	0.05	**2.67**	0.29	**2.86**	0.09
HLA-A	**1.01**	0.26	**1.63**	0.37	**1.48**	0.48	**1.48**	0.50
HLA-B	**1.00**	0.01	**1.12**	0.04	**1.05**	0.04	**1.14**	0.02
HLA-C	**1.00**	0.14	**1.31**	0.05	**1.39**	0.17	**1.38**	0.14
TAP-1	**1.01**	0.20	**1.47**	0.22	**1.87**	0.20	**2.06**	0.26
TAP-2	**1.01**	0.19	**1.45**	0.06	**1.48**	0.06	**1.66**	0.15
IFNGR1	**1.01**	0.23	**1.57**	0.06	**1.61**	0.01	**1.76**	0.17
PD-L1	**1.00**	0.09	**1.40**	0.33	**1.16**	0.08	**0.82**	0.18

FC - Fold change; ±SD - Standard deviation

Supplementary Table 2. qRT-PCR of antigen presentation genes and *PD-L1* after 48 h of treatment. $n = 3$ technical replicates.

PAKi	0 nM		5 nM		50 nM		200 nM		500 nM		1000 nM	
**Gene**	**FC**	**±SD**	**FC**	**±SD**	**FC**	**±SD**	**FC**	**±SD**	**FC**	**±SD**	**FC**	**±SD**
**A549**												
B2M	**1.00**	0.03	**1.39**	0.11	**1.72**	0.12	**1.88**	0.06	**2.03**	0.07	**1.68**	0.36
HLA-A	**1.02**	0.28	**0.54**	0.28	**0.40**	0.10	**0.76**	0.57	**1.00**	0.65	**1.03**	0.22
HLA-B	**1.01**	0.13	**1.51**	0.10	**2.80**	0.34	**3.47**	0.38	**3.76**	0.08	**3.25**	0.10
HLA-C	**1.00**	0.11	**1.16**	0.06	**1.10**	0.13	**1.30**	0.04	**1.49**	0.06	**1.36**	0.02
TAP1	**1.00**	0.11	**1.49**	0.05	**2.59**	0.18	**3.26**	0.27	**3.83**	0.24	**3.87**	0.19
TAP2	**1.00**	0.09	**1.24**	0.11	**1.49**	0.08	**1.73**	0.05	**1.84**	0.05	**1.83**	0.17
IFNGR1	**1.00**	0.07	**0.91**	0.06	**0.88**	0.02	**1.14**	0.10	**1.25**	0.10	**1.13**	0.08
PD-L1	**1.01**	0.15	**1.34**	0.03	**2.99**	0.13	**4.82**	0.06	**4.96**	0.11	**4.02**	0.73
**PC3**												
B2M	**1.02**	0.23	**1.24**	0.08	**1.35**	0.15	**1.96**	0.22	**2.12**	0.13	**2.09**	0.02
HLA-A	**1.00**	0.06	**0.72**	0.24	**1.00**	0.08	**1.15**	0.69	**0.59**	0.08	**0.58**	0.17
HLA-B	**1.00**	0.10	**0.94**	0.07	**0.74**	0.02	**1.07**	0.17	**1.06**	0.03	**1.13**	0.07
HLA-C	**1.01**	0.20	**0.99**	0.04	**0.93**	0.04	**1.03**	0.19	**1.06**	0.04	**1.07**	0.07
TAP1	**1.00**	0.09	**1.06**	0.09	**1.60**	0.10	**2.39**	0.27	**2.92**	0.27	**3.54**	0.32
TAP2	**1.00**	0.10	**1.22**	0.07	**1.23**	0.10	**1.55**	0.20	**1.73**	0.14	**2.00**	0.15
IFNGR1	**1.03**	0.26	**1.04**	0.07	**1.38**	0.19	**2.12**	0.20	**3.01**	0.45	**2.94**	0.31
PD-L1	**1.00**	0.03	**1.19**	0.07	**1.90**	0.07	**4.47**	0.71	**5.21**	0.53	**6.56**	0.87
**HT-29**												
B2M	**1.00**	0.05	**1.41**	0.05	**2.63**	0.04	**2.37**	0.03	**3.17**	0.03	**3.00**	0.92
HLA-A	**1.01**	0.17	**0.55**	0.17	**0.77**	0.11	**1.13**	0.29	**1.10**	0.07	**1.34**	0.39
HLA-B	**1.00**	0.08	**1.00**	0.11	**1.46**	0.14	**1.62**	0.39	**3.01**	0.06	**2.66**	0.24
HLA-C	**1.00**	0.07	**1.08**	0.06	**1.31**	0.05	**1.33**	0.03	**1.57**	0.44	**2.11**	0.10
TAP1	**1.00**	0.07	**1.20**	0.06	**1.33**	0.05	**1.62**	0.05	**2.83**	0.07	**4.05**	0.33
TAP2	**1.00**	0.04	**1.42**	0.07	**1.50**	0.12	**1.54**	0.09	**2.46**	0.02	**2.32**	0.23
IFNGR1	**1.00**	0.09	**0.88**	0.03	**1.59**	0.03	**1.99**	0.04	**3.18**	0.13	**4.80**	0.42
PD-L1	**1.00**	0.06	**1.33**	0.09	**2.18**	0.18	**2.44**	0.38	**3.12**	0.18	**2.37**	0.15

FC - Fold change; ±SD - Standard deviation

Supplementary Table 3. qRT-PCR analysis of type I interferon signaling genes after 48 h of treatment. $n = 3$ technical replicates.

PAKi	0 nM		2 or 50 nM		50 or 200 nM		500 nM	
**Gene**	**FC**	**±SD**	**FC**	**±SD**	**FC**	**±SD**	**FC**	**±SD**
**MCF7 (PAKi: 0, 50, 200, 500 nM)**								
IFNB1	**1.01**	0.20	**4.82**	0.95	**13.57**	0.73	**78.52**	3.52
IFNA2	**1.03**	0.43	**5.88**	1.29	**7.94**	3.88	**9.44**	2.12
IFI27	**1.02**	0.35	**1.14**	0.09	**2.38**	0.15	**3.62**	0.08
IFI44	**1.05**	0.57	**1.74**	0.14	**5.68**	0.11	**19.22**	0.58
IFI44L	**1.02**	0.37	**1.30**	0.06	**4.01**	0.07	**15.86**	0.77
**MDA-MB-468 (PAKi: 0, 50, 200, 500 nM)**								
IFNB1	**1.01**	0.19	**1.81**	0.16	**1.39**	0.37	**1.72**	0.20
IFNA2	**1.02**	0.39	**1.30**	0.32	**2.70**	0.41	**2.24**	0.29
IFI27	**1.03**	0.43	**2.76**	0.44	**3.20**	0.40	**4.61**	0.61
IFI44	**1.00**	0.14	**0.15**	0.01	**0.09**	0.01	**0.08**	0.01
IFI44L	**1.02**	0.36	**0.36**	0.06	**0.50**	0.08	**0.30**	0.11
**MEL-526 (PAKi: 0, 2, 50, 500 nM)**								
IFNB1	**1.00**	0.11	**0.93**	0.06	**18.07**	0.87	**2.43**	0.48
IFNA2	**1.04**	0.47	**0.83**	0.04	**30.98**	3.04	NA	
IFI27	**1.04**	0.46	**2.10**	0.67	**11.87**	0.84	NA	
IFI44	**1.01**	0.28	**1.54**	0.18	**4.18**	0.24	NA	
IFI44L	**1.00**	0.12	**1.30**	0.52	**23.44**	1.59	NA	
**MEL-888 (PAKi: 0, 50, 200, 500 nM)**								
IFNB1	**1.02**	0.33	**0.99**	0.31	**0.93**	0.29	**1.26**	0.16
IFNA2	**1.31**	1.30	**2.18**	1.09	**4.05**	2.32	**5.93**	1.69
IFI27	**1.00**	0.03	**2.53**	0.37	**3.27**	0.54	**3.58**	0.27
IFI44	**1.01**	0.29	**0.56**	0.08	**0.45**	0.16	**0.55**	0.20
IFI44L	**1.10**	0.79	**0.22**	0.08	**0.54**	0.39	**0.36**	0.17

FC - Fold change; ±SD - Standard deviation

Supplementary Table 4. qRT-PCR analysis of type I interferon signaling genes after 48 h of treatment. $n = 3$ technical replicates.

PAKi	0 nM		5 nM		50 nM		200 nM		500 nM		1000 nM	
**Gene**	**FC**	**±SD**	**FC**	**±SD**	**FC**	**±SD**	**FC**	**±SD**	**FC**	**±SD**	**FC**	**±SD**
**A549**												
IFNB1	**1.01**	0.14	**1.21**	0.20	**2.63**	0.36	**3.01**	0.37	**5.28**	0.89	**3.17**	0.36
IFNA2	**1.00**	0.09	**1.67**	0.74	**6.00**	1.14	**6.97**	1.45	**13.15**	2.32	**5.74**	0.98
IFI27	**1.01**	0.13	**2.04**	0.21	**2.86**	0.17	**3.42**	0.64	**3.52**	0.18	**3.88**	0.32
IFI44	**1.00**	0.10	**0.68**	0.17	**0.65**	0.14	**0.52**	0.18	**0.58**	0.16	**0.50**	0.19
IFI44L	**1.01**	0.21	**0.99**	0.52	**0.90**	0.45	**0.93**	0.65	**1.65**	0.57	**1.01**	0.87
**PC3**												
IFNB1	**1.01**	0.15	**0.96**	0.12	**1.90**	0.43	**2.57**	0.25	**3.09**	0.44	**3.55**	0.59
IFNA2	**1.01**	0.15	**1.13**	0.15	**1.38**	0.28	**2.39**	0.55	**3.18**	0.26	**1.87**	0.33
IFI27	**1.00**	0.04	**0.84**	0.09	**1.17**	0.20	**2.03**	0.25	**2.04**	0.37	**2.49**	0.30
IFI44	**1.00**	0.07	**0.87**	0.02	**0.23**	0.01	**0.07**	0.01	**0.07**	0.01	**0.08**	0.01
IFI44L	**1.00**	0.05	**0.88**	0.07	**0.43**	0.02	**0.28**	0.01	**0.26**	0.08	**0.27**	0.02
**HT-29**												
IFNB1	**1.01**	0.17	**0.46**	0.26	**1.00**	0.24	**1.16**	0.25	**1.36**	0.20	**2.30**	0.22
IFNA2	**1.01**	0.17	**1.41**	0.16	**2.77**	0.06	**3.26**	0.56	**2.86**	0.29	**7.72**	1.14
IFI27	**1.00**	0.03	**0.79**	0.05	**1.23**	0.04	**1.01**	0.02	**1.91**	0.12	**1.86**	0.12
IFI44	**1.00**	0.08	**0.50**	0.03	**0.26**	0.02	**0.22**	0.01	**0.37**	0.03	**0.27**	0.01
IFI44L	**1.00**	0.08	**0.24**	0.03	**0.09**	0.01	**0.08**	0.03	**0.25**	0.05	**0.28**	0.01

FC - Fold change; ±SD - Standard deviation

Supplementary Table 5. Sequences of primers used for qPCR.

Target	Forward (5' --> 3')	Reverse (5' --> 3')
GAPDH	GGTGAAGGTCGGAGTCAACG	TGGGTGGAATCATATTGGAACA
B2M	ATGTCTCGCTCCGTGGCCTT	GACTTTCCATTCTCTGCTGG
HLA-A	AAAAGGAGGGAGTTACACTCAGG	GCTGTGAGGGACACATCAGAG
HLA-B	CTACCCTGCGGAGATCA	ACAGCCAGGCCAGCAACA
HLA-C	CACACCTCTCCTTTGTGACTTCAA	CCACCTCCTCACATTATGCTAACA
TAP-1	GCTGTTCCTGGTCCTGGTGG	TTTCGAGTGAAGGTATCGGC
TAP-2	CAATAGCAGCGGAGAAGGTG	CTCGGCCCCAAAACTGCGAA
IFNGR1	GTCAGAGTTAAAGCCAGGGTTG	CTTCCTGCTCGTCTCCATTTAC
PD-L1	CTACTGGCATTTGCTGAACG	GACAATTAGTGCAGCCAGGT
CXCL10	GCTGCCTTATCTTTCTGACT	GGACAAAATTGGCTTGCAGG
IFNB1	CTTGGATTCCTACAAAGAAGCAGC	TCCTCCTTCTGGAACTGCTGCA
IFNA2	TGGGCTGTGATCTGCCTCAAAC	CAGCCTTTTGGAACTGGTTGCC
IFI27	CGTCCTCCATAGCAGCCAAGAT	ACCCAATGGAGCCCAGGATGAA
IFI44	GTGAGGTCTGTTTTCCAAGGGC	CGGCAGGTATTTGCCATCTTTCC
IFI44L	TGCACTGAGGCAGATGCTGCG	TCATTGCGGCACACCAGTACAG
MAVS	ATGGTGCTCACCAAGGTGTCTG	TCTCAGAGCTGCTGTCTAGCCA
TRAF6	CAATGCCAGCGTCCCTTCCAAA	CCAAAGGACAGTTCTGGTCATGG
MT-CO1	ACGTTGTAGCCCACTTCCAC	TGGCGTAGGTTTGGTCTAGG
MT-ND5	TCGAAACCGCAAACATATCA	CAGGCGTTTAATGGGGTTTA
MT-ND6	CCAATAGGATCCTCCCGAAT	AGGTAGGATTGGTGCTGTGG
MT-CYB	AGACAGTCCCACCCTCACAC	GGTGATTCCTAGGGGGTTGT
