# Supplementary material for: Systematic investigation of chemo-immunotherapy synergism to shift anti-PD-1 resistance in cancer
Source: Nat Commun. 2024 Apr 12;15:3178. doi: 10.1038/s41467-024-47433-y (PMC11015024; doi:10.1038/s41467-024-47433-y)
Supplement: Supplementary file 3 — Description of Additional Supplementary Files [file 41467_2024_47433_MOESM3_ESM.pdf]

Description of Additional Supplementary Files

Supplementary Data 1. Treatment-induced expression change profiling can predict anti-PD-1 response in patients.

Supplementary Data 2. Genes involved in R and S signatures are highly correlated with patient prognosis and immune responses.

Supplementary Data 3. Genetic inhibition of genes in R and S signature can shift immunotherapy response phenotypes.

Supplementary Data 4. Shift ability analysis on compound-treated transcriptomes characterized chemo-immunotherapy synergism.

Supplementary Data 5. Integrating shift ability analysis on genetic and pharmacological inhibition identified novel compounds that can sensitize anti-PD-1 response.

Supplementary Data 6. Mechanism of chemo-immunotherapy synergisms.
