# Supplementary material for: Systematic investigation of chemo-immunotherapy synergism to shift anti-PD-1 resistance in cancer
Source: Nat Commun. 2024 Apr 12;15:3178. doi: 10.1038/s41467-024-47433-y (PMC11015024; doi:10.1038/s41467-024-47433-y)
Supplement: Supplementary file 10 — Reporting Summary [file 41467_2024_47433_MOESM10_ESM.pdf]

Corresponding author(s): Da Yang
Last updated by author(s): Mar 22, 2024

Reporting Summary

Nature Portfolio wishes to improve the reproducibility of the work that we publish. This form provides structure for consistency and transparency in reporting. For further information on Nature Portfolio policies, see our Editorial Policies and the Editorial Policy Checklist.

Statistics

For all statistical analyses, confirm that the following items are present in the figure legend, table legend, main text, or Methods section.

n/a	Confirmed	
☐	☒	The exact sample size (*n*) for each experimental group/condition, given as a discrete number and unit of measurement
☐	☒	A statement on whether measurements were taken from distinct samples or whether the same sample was measured repeatedly
☐	☒	The statistical test(s) used AND whether they are one- or two-sided<br>*Only common tests should be described solely by name; describe more complex techniques in the Methods section.*
☐	☒	A description of all covariates tested
☐	☒	A description of any assumptions or corrections, such as tests of normality and adjustment for multiple comparisons
☐	☒	A full description of the statistical parameters including central tendency (e.g. means) or other basic estimates (e.g. regression coefficient) AND variation (e.g. standard deviation) or associated estimates of uncertainty (e.g. confidence intervals)
☐	☒	For null hypothesis testing, the test statistic (e.g. *F*, *t*, *r*) with confidence intervals, effect sizes, degrees of freedom and *P* value noted<br>*Give P values as exact values whenever suitable.*
☒	☐	For Bayesian analysis, information on the choice of priors and Markov chain Monte Carlo settings
☒	☐	For hierarchical and complex designs, identification of the appropriate level for tests and full reporting of outcomes
☐	☒	Estimates of effect sizes (e.g. Cohen's *d*, Pearson's *r*), indicating how they were calculated

Our web collection on statistics for biologists contains articles on many of the points above.

Software and code

Policy information about availability of computer code

Data collection	No software is used.
Data analysis	For difference comparison, if not being particularly specified, Wilcoxon rank-sum test was applied to compare the differences between two unpaired groups; Wilcoxon signed rank test was applied to compare the differences between two paired groups; one-way ANOVA was applied to compare the differences between three or more groups; Kolmogorov-Smirnov test was applied to compare the differences between two continuous distributions. For correlation analysis, both Pearson and Spearman's correlation were applied in order to avoid the potential conflicts on linearity assumption. For enrichment and exclusiveness, pre-rank Gene Set Enrichment Analysis was applied to assess the enrichment of specific features in single samples; hypergeometric test was applied for between-group comparison. For survival analysis, both Cox Proportional Hazard model and log rank test were utilized to compare prognosis between groups. All the computational and statistical analyses presented in this study were implemented by Python (version 3.8.0) in local or on the cluster of University of Pittsburgh Center for Research Computing (CRC).<br><br>GrapPad Prism (10.1.2,GraphPad) is used to analyze in vitro and in vivo data.<br><br>The source code in this study for shift ability analysis is available at https://github.com/DaYangLab2015/ChemoImmunoSyng.

For manuscripts utilizing custom algorithms or software that are central to the research but not yet described in published literature, software must be made available to editors and reviewers. We strongly encourage code deposition in a community repository (e.g. GitHub). See the Nature Portfolio guidelines for submitting code & software for further information.

Data

Policy information about availability of data

All manuscripts must include a data availability statement. This statement should provide the following information, where applicable:

- Accession codes, unique identifiers, or web links for publicly available datasets
- A description of any restrictions on data availability
- For clinical datasets or third party data, please ensure that the statement adheres to our policy

Post-perturbation cell line transcriptome data, including shRNA and compound treatment, were collected from the Expanded Connectivity Map (CMAP) LINCS Resource 2020 (complete version, 11/23/2021) through the CLUE portal (http://clue.io). Gene expression and clinical data of patients treated with immune checkpoint blockade were collected from Gene Expression Omnibus (GEO) with accession number GSE91061, GSE168204, GSE115821 and GSE93157, from European Nucleotide Archive (ENA) with accession number PRJEB23709, and from MTA transfer with University of California, Los Angeles (UCLA) with accession ID DUA00004062. Gene expression and clinical data of The Cancer Genome Atlas (TCGA) patients were obtained from the GDC data portal (http://portal.gdc.cancer.gov). The authors declare that all code and data needed to evaluate the conclusions in the paper are present in the paper, code repository (https://github.com/DaYangLab2015/ChemoImmunoSyng) and/or the Supplementary Materials.

Research involving human participants, their data, or biological material

Policy information about studies with human participants or human data. See also policy information about sex, gender (identity/presentation), and sexual orientation and race, ethnicity and racism.

Reporting on sex and gender	N/A
Reporting on race, ethnicity, or other socially relevant groupings	N/A
Population characteristics	N/A
Recruitment	N/A
Ethics oversight	N/A

Note that full information on the approval of the study protocol must also be provided in the manuscript.

Field-specific reporting

Please select the one below that is the best fit for your research. If you are not sure, read the appropriate sections before making your selection.

☒ Life sciences ☐ Behavioural & social sciences ☐ Ecological, evolutionary & environmental sciences

For a reference copy of the document with all sections, see nature.com/documents/nr-reporting-summary-flat.pdf

Life sciences study design

All studies must disclose on these points even when the disclosure is negative.

Sample size	The exact numbers of samples (n) used in this study are indicated in the respective Figure legends.
Data exclusions	No data was excluded.
Replication	The number of replicates has been stated in the Figure legends. Replicated experiments were successful. In vivo studies were conducted one time with n=5 or 6 mice per group.
Randomization	For in vivo studies, after tumor engraftment mice were randomly divided into groups and treated. For all in vitro experiments, all cell lines were treated in identical condition and dosages of drugs used were stated in Figure legends.
Blinding	For in vitro experiments, blinding was not possible as the same the researcher treated the cells and performed the analysis. However, all analyses within experiments conducted performed in identical manner, prior knowledge of treatment had no impact on output results.

Reporting for specific materials, systems and methods

We require information from authors about some types of materials, experimental systems and methods used in many studies. Here, indicate whether each material, system or method listed is relevant to your study. If you are not sure if a list item applies to your research, read the appropriate section before selecting a response.

Materials & experimental systems

n/a	Involved in the study
	[x] Antibodies
	[x] Eukaryotic cell lines
[x]	Palaeontology and archaeology
	[x] Animals and other organisms
[x]	Clinical data
[x]	Dual use research of concern
[x]	Plants

Methods

n/a	Involved in the study
[x]	ChIP-seq
	[x] Flow cytometry
[x]	MRI-based neuroimaging

Antibodies

Antibodies used

Antibodies used for western blotting:
anti-LC3A/B (D3U4C, Cell Signaling Technology, Cat.# 12741), dilution: 1:1000
anti-STAT1 (D1K9Y, Cell Signaling Technology, Cat.# 14994), dilution: 1:1000
anti-phospho-STAT1 (D4A7, Cell Signaling Technology, Cat.# 7649), dilution: 1:1000
anti-STING (D2P2F, Cell Signaling Technology, Cat.# 13647), dilution: 1:1000
anti-phospho-STING (E9A9K, Cell Signaling Technology, Cat.# 50907), dilution: 1:1000
anti-MAVS (Cell Signaling Technology, Cat.# 3993), dilution: 1:1000
anti-GAPDH (6C5, Sigma-Aldrich, Cat.# MAB374), dilution: 1:1000
anti-β-actin (AC-15, Sigma-Aldrich, Cat.# A5441), dilution: 1:20,000
Goat anti-mouse IgG HRP conjugated secondary antibodies (Santa Cruz Biotech, Cat.# SC-2005)
Goat anti-Rabbit IgG HRP conjucated secondary antibody (Santa Cruz Biotech, Cat.# SC-2004)

Antibodies used for Flow cytometry:
Zombie NIR (Biolegend, Cat.# 423105), dilution: 1/1000
PerCP anti-mouse CD45 Antibody (30-F11, Biolegend, Cat.# 103130), dilution: 1/200
Brilliant Violet 737 anti-mouse CD4 Antibody (GK1.5, BD Biosciences, Cat.# 612761), dilution: 1/200
PE-Cy7 anti-mouse IFN-γ Antibody (XMG1.2, BD Biosciences, Cat.# 557649), dilution: 1/200
Brilliant Violet 615 anti-mouse CD279 (PD-1) Antibody (RMP1-30, BD Biosciences, Cat.# 752354), dilution: 1/200
APC anti-mouse CD11b Antibody (M1/70, eBioscience, Cat.# 17-0112-81), dilution: 1/200
Brilliant Violet 510 anti-mouse Ly-6G/Ly-6C (Gr-1) Antibody (RB6-8C5, Biolegend, Cat.# 108457), dilution: 1/200
Pacific Blue MHCII anti-mouse Antibody (M5/114.15.2, Biolegend, Cat.# 107620), dilution: 1/200
APC/Cyanine7 anti-mouse F4/80 Antibody (BM8, Biolegend, Cat.# 123118), dilution: 1/200
FITC anti-mouse CD206 (MMR) Antibody (MR5D3, Invitrogen, Cat.# MA5-16870), dilution: 1/200
PE anti-mouse CD163 Antibody (S15049I, Biolegend, Cat.# 155307), dilution: 1/200
Anti-dsRNA (J2) antibody (J2, Cell Signaling Technology, Cat.# 76651), dilution: 2.5 μg/mL
Alexa Fluor 488 conjugated goat anti-mouse IgG H&L antibody (abcam, Cat.# ab150113), dilution: 2.2 μg/mL

Antibodies used for immunofluorescence staining:
Anti-dsRNA (J2) antibody (J2, Cell Signaling Technology, Cat.# 76651), dilution: 2.5 μg/mL
Alexa Fluor 488 conjugated goat anti-mouse IgG H&L antibody (abcam, Cat.# ab150113), dilution: 2 μg/mL

Validation

All antibodies were used were based on the recommendation from the manufacturer's website or previously published study that used similar application.

Anti-dsRNA (J2) antibody - Dhir, A., Dhir, S., Borowski, L.S. et al. Mitochondrial double-stranded RNA triggers antiviral signalling in humans. Nature 560, 238–242 (2018). https://doi.org/10.1038/s41586-018-0363-0

Zombie NIR (Biolegend, Cat# 423105, https://www.biolegend.com/fr-lu/products/zombie-nir-fixable-viability-kit-8657)
PerCP anti-mouse CD45 Antibody (Biolegend, Cat# 103130, https://www.biolegend.com/fr-fr/products/percp-anti-mouse-cd45-antibody-4265)
Brilliant Violet 737 anti-mouse CD4 Antibody (BD Biosciences, Cat# 612761, https://www.bdbiosciences.com/en-us/products/reagents/flow-cytometry-reagents/research-reagents/single-color-antibodies-ruo/buv737-rat-anti-mouse-cd4.612761)
PE-Cy7 anti-mouse IFN-γ Antibody (BD Biosciences, Cat# 557649, https://www.bdbiosciences.com/en-us/products/reagents/flow-cytometry-reagents/research-reagents/single-color-antibodies-ruo/pe-cy-7-rat-anti-mouse-ifn.557649)
Brilliant Violet 615 anti-mouse CD279 (PD-1) Antibody (BD Biosciences, Cat# 752354, https://www.bdbiosciences.com/en-us/products/reagents/flow-cytometry-reagents/research-reagents/single-color-antibodies-ruo/buv615-rat-anti-mouse-cd279-pd-1.752354)
APC anti-mouse CD11b Antibody (eBioscience, Cat# 17-0112-81, https://www.thermofisher.com/antibody/product/CD11b-Antibody-clone-M1-70-Monoclonal/17-0112-81)
Brilliant Violet 510 anti-mouse Ly-6G/Ly-6C (Gr-1) Antibody (Biolegend, Cat# 108457, https://www.biolegend.com/en-us/products/brilliant-violet-510-anti-mouse-ly-6g-ly-6c-gr-1-antibody-8614)
Pacific Blue MHCII anti-mouse Antibody (Biolegend, Cat#, 107620, https://www.biolegend.com/en-us/products/pacific-blue-anti-mouse-i-a-i-e-antibody-3136)

APC/Cyanine7 anti-mouse F4/80 Antibody (Biolegend, Cat# 123118, https://www.biolegend.com/en-us/products/apc-cyanine7-anti-mouse-f4-80-antibody-4072)
FITC anti-mouse CD206 (MMR) Antibody (Invitrogen, Cat# MA5-16870, https://www.thermofisher.com/antibody/product/CD206-Antibody-clone-MR5D3-Monoclonal/MA5-16870)
PE anti-mouse CD163 Antibody (Biolegend, Cat#155307, https://www.biolegend.com/en-us/products/pe-anti-mouse-cd163-antibody-18223)

Eukaryotic cell lines

Policy information about cell lines and Sex and Gender in Research

Cell line source(s)

Human breast cancer cell lines MCF7 and MDA-MB-468, human melanoma cancer cell lines MEL-526 and MEL-888, human lung carcinoma cell line A549, human prostate cancer cell line PC3, human colorectal adenocarcinoma cell line HT-29, murine melanoma cell line B16, murine colorectal cancer cell line CT26, and murine prostate cancer cell line MyC-CaP were purchased from American Type Culture Collection (ATCC)

MAVS knockout MCF7 cells:
We designed three guide RNA (gRNA) for MAVS and one scrambled gRNA as a control and used lentiCRISPRv2 vector. Lentiviral particles were prepared after transfection of plasmids into HEK-293T cells using Lipofectamine 2000™ (Invitrogen, #11668019). Targeted cells were infected with the lentivirus packaged by Cas9 and single-guide RNA (sgRNA) expression plasmid encoding puromycin resistance (Addgene plasmid, #52961). Guide RNA sequences used to generate MAVS knockout:
gMAVS_F1: caccgCTTCCGGTCGGCTTGTGGCC;
gMAVS_R1: aaacGGCCACAAGCCGACCGGAAGc;
gMAVS_F2: caccgAGGTGGCCCGCAGTCGATCC;
gMAVS_R2: aaacGGATCGACTGCGGGCCACCTc;
gMAVS_F3: caccgGTGTCTTCCAGGATCGACTG;
gMAVS_R3: aaacCAGTCGATCCTGGAAGACACc

Authentication

Not performed for MCF7, MDA-MB-468, MEL-526, MEL-888, A549, PC3, HT-29, B16, CT26, and MyC-CaP cell lines.

MAVS knockout MCF7 cells:
The knockout efficiency was determined by the immunoblotting analysis of MAVS after selection of puromycin resistance cells. gMAVS_F3/R3 exhibited the maximum knockout efficiencies of MAVS gene in MCF7 cells and used in this study.

Mycoplasma contamination

Cell lines were tested negative for Mycoplasma contamination using Homemade PCR test for Mycoplasma contamination test.
Primers used:
Forward Primers pool:
Myco-5-1 CGCCTGAGTAGTACGTTCGC
Myco-5-2 CGCCTGAGTAGTACGTACGC
Myco-5-2 TGCCTGAGTAGTACATTCGC
Myco-5-2 TGCCTGGGTAGTACATTCGC
Myco-5-5 CGCCTGGGTAGTACATTCGC
Myco-5-6 CGCCTGAGTAGTATGCTCGC
Reverse primers pool:
Myco-3-1 GCGGTGTGTACAAGACCCGA
Myco-3-2 GCGGTGTGTACAAAACCCGA
Myco-3-3 GCGGTGTGTACAAACCCCGA

Commonly misidentified lines
(See ICLAC register)

This study did not use commonly misidentified lines.

Animals and other research organisms

Policy information about studies involving animals; ARRIVE guidelines recommended for reporting animal research, and Sex and Gender in Research

Laboratory animals

Female BALB/c mice, C57BL/6 mice, and FVB/NJ mice aged between 4–6 weeks were purchased from The Jackson Laboratories (CT, USA). Mice were housed under pathogen-free conditions according to AAALAC (Association for Assessment and Accreditation of Laboratory Animal Care) guidelines. The mouse-related experiments were performed in full compliance with institutional guidelines and approved by the Animal Use and Care Administrative Advisory Committee at the University of Pittsburgh under Protocol #: 21099779. Mice were housed at an ambient temperature of 22 °C (22–24 °C) and humidity of 45%, with a 14/10 day/night cycle (on at 6:00, off at 20:00), and allowed access to food ad libitum.

Wild animals

The study did not involve wild animals.

Reporting on sex

Indicate if findings apply to only one sex; describe whether sex was considered in study design, methods used for assigning sex. Provide data disaggregated for sex where this information has been collected in the source data as appropriate; provide overall numbers in this Reporting Summary. Please state if this information has not been collected. Report sex-based analyses where performed, justify reasons for lack of sex-based analysis.

Field-collected samples	No Field-collected samples were used in this study
Ethics oversight	The mouse-related experiments were performed in full compliance with institutional guidelines and approved by the Animal Use and Care Administrative Advisory Committee at the University of Pittsburgh.

Note that full information on the approval of the study protocol must also be provided in the manuscript.

Flow Cytometry

Plots

Confirm that:

- [x] The axis labels state the marker and fluorochrome used (e.g. CD4-FITC).
- [x] The axis scales are clearly visible. Include numbers along axes only for bottom left plot of group (a 'group' is an analysis of identical markers).
- [ ] All plots are contour plots with outliers or pseudocolor plots.
- [x] A numerical value for number of cells or percentage (with statistics) is provided.

Methodology

Sample preparation	In vivo tumor immune profiling:<br>Flow cytometry was performed with LSRII (BD Biosciences) and Aurora (Cytek Biosciences) instruments and analyzed by FlowJo (BD Biosciences). B16 and MyC-CaP tumors were prepared for single cell suspensions. Briefly, tumors were dissected and transferred into RPMI-1640. Tumors were disrupted mechanically using scissors, digested with a mixture of deoxyribonuclease I (0.3 mg/ml, Sigma-Aldrich) and TL Liberase (0.25 mg/ml, Roche) in serum-free RPMI-1640 at 37 °C for 30 min, and dispersed through a 40 μm cell strainer (BD Biosciences). After red blood cell lysis, live/dead cell discrimination was performed using a Zombie NIR Fixable Viability Kit (BioLegend, dilution: 1/1,000) at 4 °C for 30 min in PBS. Surface staining was performed at 4 °C for 30 min in FACS staining buffer (1× phosphate-buffered saline/5% FBS/0.5% sodium azide) containing designated antibody cocktails (PerCP anti-mouse CD45 antibody, Brilliant Violet 737 anti-mouse CD4 antibody, Brilliant Violet 615 anti-mouse PD-1 antibody, APC anti-mouse CD11b antibody, Brilliant Violet 510 anti-mouse Gr-1 antibody, APC/Cyanine7 anti-mouse F4/80 antibody, Pacific Blue anti-mouse MHC II antibody and PE anti-mouse CD163 antibody; dilution: 1/200 for all antibodies). For intracellular protein staining (FITC anti-mouse CD206 antibody; dilution: 1/200 for antibody), cells were fixed and permeabilized using the BD Cytofix/Cytoperm kit, following the manufacturer's instructions. For intracellular cytokine staining (PE-Cy7 anti-mouse IFN-γ antibody; dilution: 1/200 for antibody), cells were stimulated with phorbol 12-myristate-13-acetate (100 ng/mL) and ionomycin (500 ng/mL) for 6 h in the presence of Monensin. Cells were fixed/permeabilized using the BD Cytofix/Cytoperm kit before cell staining.<br><br>Flow cytometry detection of mitophagy: Cancer cells cultured in 6 well plates and washed two times with HBSS and incubated with Mitophagy dye at 37°C for 30 min. After two washes cells, cells were incubated with or without drugs for 24 h. Then cells were collected by trypsinization, washed twice with HBSS and incubated with Lyso dye at 37°C for 30 min. After two washes cells were suspended in HBSS and analyzed using MACSQuant analyzer (Miltenyi Biotec). Blue 655-730 nm (corresponds to PerCP-Cy5.5) and violet 525/50 nm (corresponds to VioGreen) fluorescence filters were used for Mitophagy dye and Lyso dye, respectively. Centrifugation steps were performed at 200 x g in RT. Data were analyzed using FlowJo Software (10.9.0).<br><br>Flow cytometry analysis of dsRNA: After 24 h treatment, cells were collected by trypsinization and washed twice with PBS. Cells were fixed with 4% formaldehyde for 20 min at RT. After two washes with PBS, cells were permeabilized for 15 min at RT using 0.1% Triton X-100 in PBS. Cells then incubated with 1% BSA for at RT for 1 h followed by incubation with 2.5 μg/mL of anti-dsRNA (J2) antibody (CST, #76651) at RT for 1 h. After three washed cells were incubated with 2.2 μg/mL of Alexa Fluor 488 (AF488) conjugated goat anti-mouse IgG H&L antibody (abcam, #ab150113) at RT for 1 h. Cells then washed three times with PBS and suspended in 0.5% BSA in PBS. Centrifugation steps were performed at 300 x g in 4°C. Cells were analyzed using MACSQuant analyzer (Miltenyi Biotec). Data were analyzed using FlowJo Software (10.9.0).
Instrument	LSRII (BD Biosciences) and Aurora (Cytek Biosciences) flow cytometers were used for in vivo tumor immune profiling.<br>MACSQuant 10 analyzer (Miltenyi Biotec) used for mitophagy and dsRNA detection analyses.
Software	BD FACSDiva Software (BD Biosciences) , SpectroFlo (Cytek Biosciences) and FlowJo Software (FlowJo) used for tumor samples immune profiling.<br>MACSQuantify Software and FlowJo Software used for dsRNA and mitophagy detection analyses.
Cell population abundance	For in vitro experiments, after gating selection of single cell population 45-80% of total starting cell population taken.<br>For in vivo tumor immune profiling, cell populations of interest were collected as much as possible in the intratumoural immunology assay.

Gating strategy

For in vivo tumor immune profiling, T cells were gated under Zombie NIR- & CD45+ cells. CD4 cell were gated under T cells. IFNγ+ and PD-1+ cells were gated under CD4+ cells. Macrophage population were characterized by using Gr-1-, CD11b+, MHCII+ and F4/80 gating. CD163+ macrophages and M2-like (CD206+) were gated under macrophage populations.
For all in vitro experiments, cells were gated to select singlets events (i.e., FSC-H x FSC-A followed by SSC-A x FSC-A) and then plotted to analyze mitophagy and dsRNA.

☐ Tick this box to confirm that a figure exemplifying the gating strategy is provided in the Supplementary Information.
